# Assessing failure patterns of radical intent radiation strategies in patients with locally advanced carcinoma of the esophagus

**DOI:** 10.1002/cnr2.1332

**Published:** 2020-12-28

**Authors:** Shagun Mishra, Farhan Ahmad, Shalini Singh, Rajneesh K. Singh, Koilpillai J. Maria Das, Shaleen Kumar

**Affiliations:** ^1^ Department of Radiotherapy Sanjay Gandhi Postgraduate Institute of Medical Sciences Lucknow Uttar Pradesh India; ^2^ Department of Gastrosurgery Sanjay Gandhi Postgraduate Institute of Medical Sciences Lucknow Uttar Pradesh India

**Keywords:** carcinoma of the esophagus, chemoradiation, dose escalation, failure patterns, radiation fields

## Abstract

**Background:**

Patterns of failure following definitive CRT (dCRT) are different as compared to neoadjuvant chemoradiotherapy (NACRT) with increased locoregional failures documented with dCRT.

**Aim:**

To document failure patterns in patients with esophageal carcinoma treated with neoadjuvant and definitive intent radiation strategies.

**Methods:**

Subjects were 123 patients treated with two chemoradiotherapy strategies. Group 1 (n = 99) underwent dose escalated definitive chemoradiotherapy (dCRT), Group 2 (n = 24) received neoadjuvant chemoradiotherapy (NACRT) followed by surgery. Cumulative incidence of locoregional failure (LRF), local failure (LF), regional lymph node failure (RLNF), and distant metastasis (DM) were computed; differences between the groups was evaluated using log rank test. Univariable and multivariable predictors of failure were identified using Cox regression analysis.

**Results:**

Cumulative LRF: 64% in Group 1 vs 35% in Group 2 (*P* = .050). Cumulative LF: 59% in Group 1 vs 12% in Group 2 (*P* = .000). Cumulative RLNF: 30% in Group 1 vs 24% in Group 2 (*P* = .592). Most common RLNF: mediastinum for both groups (6% vs 12.5%, respectively). Distant metastasis: 40.4% Group 1 vs 17% Group 2 (*P* = .129), predominantly lung (Group 1, 5%), and nonregional nodes (Group 2, 8.3%). Univariate analysis identified age ≤50, absence of concurrent chemotherapy, dose ≤50 Gy, and incomplete radiotherapy to predict higher odds of LRF and DM for Group 1; absence of comorbidities predicted for lower odds of LRF for Group 2. Age ≤50 predicted for higher odds of RNLR for Group 1, while absence of comorbidities predicted for lower odds of RNLR in Group 2. Multivariate analysis identified age ≤50, incomplete radiotherapy, and absence of concurrent chemotherapy to predict higher odds of LRF for Group 1. Age ≤50, absence of concurrent chemotherapy predicted higher odds of DM for Group 1. Absence of comorbidity predicted lower odds of LRF in Group 2.

**Conclusion:**

LRF is common in both groups, with LF being predominant in dCRT as opposed to RNLF in NACRT. Age ≤50, absence of concurrent chemotherapy is a predictor of LRF and DM in dCRT.

## 1 INTRODUCTION

Chemoradiotherapy (CRT) plays an important role in the management of esophageal carcinoma (EC) in the neoadjuvant setting for operable patients, and as a definitive treatment for those who are not resectable due to medical or technical considerations.^1^, ^2^ Patient choice and available expertise can have an impact on decision making. On the Indian subcontinent, where squamous cell carcinoma (SCC) is the predominant histology, probably fewer than 10% of patients would be candidates for resection (i.e., disease in the middle or lower third, with regional spread alone), and after excluding those with medical or other reasons for inoperability, about a third would not proceed to surgery.^3^ National practice patterns in Western countries have suggested more frequent use of definitive chemoradiotherapy (dCRT) than neoadjuvant CRT (NACRT) followed by surgery.^4^

While CRT plays an important role in the treatment of EC, there is still no evidence‐based consensus on the accurate definition of the tumor volume delineation in esophageal cancer. Studies of dCRT and NACRT have used variable definitions of elective lymph node irradiation (ELNI) and clinical target volume (CTV). The National Comprehensive Cancer Network guidelines recommend that CTV should include the areas at risk for microscopic disease as well as elective nodal regions, depending on the location of the primary tumor in the esophagus.^5^ The choice of elective nodal regions depends on the probability of lymph node involvement as understood from patterns of failure and surgical series.^6^, ^7^, ^8^ However, still there are no consistent standards for constructing proper CTV worldwide.^9^ For both dCRT and NACRT, similar principles of tumor volume delineation are applied. Different methods and opinions have been reported and practiced in various countries.^10^ Therefore, understanding relapse patterns is important, not only to gain insight into the effectiveness of the combined treatment but also to foster improvements in designing prophylactic target volumes.

The other unresolved question in esophageal cancers is the dose in the dCRT setting. Although radiation dose escalation has failed to improve local control or survival, a dose of 60 Gy is more popular in Asian countries^11^ where SCC is the predominant histological type; with modern radiotherapy (RT) techniques, the role of dose escalation is being revisited in randomized trials.^12^

This study attempts to discern the failure patterns in our population of predominantly thoracic esophageal SCC (ESCC) treated after two radical therapeutic approaches, namely escalated dose of dCRT and NACRT followed by surgery, and to help optimize the field design with particular reference to designing nodal CTV for both these approaches. We assessed a variety of patients from the perspective of their disease‐related and treatment characteristics for their potential utility as predictors of failure, reasoning that such factors may uncover opportunities for targeted intensification of therapy in patients with poor prognostic factors.

## 2 MATERIAL AND METHODS

Between January 2011 and December 2014, a total of 123 consecutively registered patients (Figure S1) were identified, who were assessed by a multidisciplinary team, and treated with radical intent CRT strategies. For NACRT‐assigned patients, those who proceeded with surgery were selected to understand the failure patterns for this group. Pretreatment workup included complete history, physical examination, routine blood and biochemical test, barium swallow test, pulmonary function test, contrast‐enhanced computed tomography (CT) of neck/chest/abdomen, and endoscopy with biopsies. The PET/CT staging was not routinely done. Clinical and pathological information was extracted from patient case files and hospital medical records. Group 1 included patients undergoing dCRT, and Group 2 comprised patients undergoing NACRT followed by surgery.

Group 1, Definitive CRT (dCRT): patients deemed unsuitable for surgery in view of medical reasons (comorbidity/performance/unresectable) or personal choice received dCRT. These patients received 60‐66 Gy/30‐33# with concurrent weekly Cisplatin 35 mg/m^2^.

Group 2, Neoadjuvant CRT (NACRT) followed by Surgery: operable and fit patients, T2‐4 ± node‐positive with performance ≥80, were selected for this approach. These patients received a dose of 45 Gy in 25 fractions with concurrent weekly Cisplatin 35 mg/m^2^. They were reassessed both clinically and radiologically with CECT scan after NACRT in a multidisciplinary clinic for surgery. The majority underwent a transthoracic resection with two‐field lymph node dissection.

### 2.1 Radiotherapy target volumes

The gross tumor volume was defined by combining information from computed tomography, esophagogastroduodenoscopy, barium scan, and included all involved local and regional lymph nodes (Figure S2). The radiotherapy clinical target volume was defined by the gross tumor volume plus a minimum of 1 cm radially and 3 cm longitudinally to a dose of 36 Gy, followed by a cone down (2 cm longitudinal margins to gross tumor volume) to a dose of 45 Gy or 60 to 66 Gy. Gross nodal disease identified as enlarged nodes on CECT scans received a dose of 45 Gy in Group 2 or 60 Gy in Group 1. In the patients with gastroesophageal junction involvement, and who were not being undertaken for surgery, the dose was restricted to 55.8 Gy in 31 fractions respecting stomach tolerance. Prophylactic nodal radiation to the supraclavicular (SCF) region was used in cases when the bulk of disease was supracarinal (primarily Group 1). Doses to the prophylactic SCF nodal volumes were 45 Gy/25#. Three‐dimensional conformal treatment was mostly used with phase I treated by AP/PA fields followed by phase II treated by three fields, both with multileaf collimator conformation.

### 2.2 Follow‐up and disease recurrence

All the patients were reviewed every 3 months for the first 3 years after dCRT or surgery, and every 6 months thereafter. The Group 1 patients were deemed to have persistence of local disease if there was a failure to respond to treatment within 3 months of the date of completion of treatment. Disease recurrence was suspected clinically (reappearance or worsening of dysphagia), and confirmed with investigations, usually by means of endoscopy, and persistence of radiological stricture on barium swallow. The CECT scans were done in all the cases undergoing surgery with suspected recurrence, whereas for dCRT, CECT at recurrence was not mandatory. Patterns of recurrence were defined as locoregional, and distant with locoregional failures (LRF) were further stratified as local failure (LF) and regional lymph node failure (RLNF). Locoregional failure was defined as a persistence of residual disease, or recurrence locally or in the regional nodal areas including the mediastinal, celiac, and supraclavicular regions irrespective of location of disease. Regional lymph node failure was defined as a disease in the mediastinal, celiac, or supraclavicular location. Distant metastasis (DM) included all nonregional nodal and visceral sites. Biopsy proof of recurrence was not mandatory. The time of recurrence was taken as the date of the confirmatory investigation, or reappearance of symptoms, whichever was earlier. The LRF was mapped as infield if located at a primary site within the planned field, or out of field recurrence if located outside the planned field.

The institutional review board approved this study and waived the requirement for written informed consent due to its retrospective nature.

### 2.3 Statistics

Demographic data and failure patterns were summarized using crude percentages. The differences between the groups were evaluated using the Pearson's Chi‐Square Test/Fisher's Test, and the Mann Whitney test for categorical and continuous variables, respectively. Cumulative incidences of locoregional, local, regional, and distant failures were calculated and compared using the log rank test. Univariate analyses, examining factors influencing local and distant control, were initially examined; those found to be significant or of clinical relevance were retained for multivariate cox regression analysis. The overall survival (OS) was calculated from the date of biopsy to death due to any cause, assuming the worst‐case scenario with all lost to follow‐up as events, using the Kaplan Meier curves and compared between groups using the log rank test. Data analysis was carried out using the Statistical Package for the Social Sciences (SPSS) version 20.

## 3 RESULTS

Between January 2011 and December 2014, a total of 123 consecutively registered patients treated with radical intent CRT strategies were identified. The median follow‐up of surviving patients was 82 months with a minimum follow‐up of 5 years. Overall, Group 1 had 99 patients, and Group 2 had 24 patients. The demographic and treatment characteristics are listed in Table 1. Generally, local failure was the prevalent mode of failure in Group 1 (seen in 48.5% of patients) as opposed to nodal failure in Group 2 (seen in 17% of patients; Table 2).

**TABLE 1:** Demographic and treatment factors

Variable	Group 1 dCRT (n = 99)	Group 2 NACRT (n = 24)	*P*‐value
*Age*: *Median (Range)*	57(31‐80)	57(28‐64)	
≤50	27(27.3%)	8(33.3%)	
>50	72(72.7%)	16(66.7%)	.616^a^
*Gender*			
Male	63(63.6%)	17(70.8%)	
Female	36(36.4%)	7(29.2%)	.635^a^
*Comorbidities*			
Yes	53(53.5%)	19(79.2%)	
No	45(45.5%)	05(20.8%)	.036^a^
*Tobacco habits*			
Yes	34(34.3%)	12(50%)	
No	65(65.7%)	12(50%)	.166^a^
*Dysphagia*			
Grade 1	22(22.2%)	2(8.3%)	
Grade 2	30(30.3%)	11(45.8%)	
Grade 3	38(38.4%)	8(33.3%)	
Grade 4	5(5.1%)	2(8.3%)	
Unknown	4(4%)	1(4.2%)	.195^a^
*Dysphagia duration (months)*			
Median (Range)	3(1–24)	3(1‐12)	.754^b^
*Weight loss*			
Absent	16(16.2%)	3(12.5%)	
Present	82(82.8%)	21(87.5%)	.763^a^
*Weight loss*			
10%	58(58.6%)	9(37.5%)	
>10%	39(39.4%)	15(62.5%)	.066^a^
*Histology*			
Squamous	96(97%)	21(87.5%)	
Adenocarcinoma	3(3%)	3(12.5%)	.088^a^
*Grade*		‐	
Well differentiated	10(10.1%)		
Moderately differentiated	29(29.3%)	10(4.2%)	.075^a^
Poorly differentiated	4(4%)	1(45.8%)	
Unknown	56(56.6%)	13(54.2%)	
*Location*			
Upper Thoracic	35(35.4%)		
Middle Thoracic	42(42.4%)	11(45.8%)	
Lower Thoracic	22(22.2%)	13(54.2%)	.000^a^
*Length*			
≤5 cm	22(22.2%)	9(37.5%)	
>5 cm	77(77.8%)	15(62.5%)	.188^a^
*T stage*			
T2	1(1%)	1(4.2%)	
T3	35(35.4%)	13(54.2%)	
T4	24(24.2%)	6(25%)	
Unknown	39(39.4%)	4(16.7%)	.576^a^
*N stage*			
N0	20(20.2%)	7(29.2%)	
N1	16(16.2%)	7(29.2%)	
N2	15(15.2%)	6(25%)	
N3	10(10.1%)	0	
Unknown	38(38.4%)	4(16.7%)	.275^a^
*RT technique*			
3DCRT	86(87%)	24(100%)	.070^a^
IMRT	13(13%)	0	
*RT dose (Gy)*			
Median (Range)	66(8‐68)	45	‐
≤50Gy	17(17%)		
>50Gy	83(83%)		
*RT complete*			
Yes	85(85.9%)	24(100%)	‐
No	14(14.1%)		
*Reasons incomplete*			‐
LFU	9	‐	
Death	4		
RT Toxicity	1		
Dose	66(8.8‐68)	45(45‐46)	‐
*NACT*			
Yes	14(14.1%)	6(25%)	
No	85(85.9%)	18(75%)	.221^a^
*Concurrent chemotherapy*			
Yes	68(68.7%)	23(95.8%)	
No	29(29.3%)	1(4.2%)	.008^a^
*Surgery type*	‐		‐
Transthoracic		14(58%)	
Transhiatal		10(42%)	
*Pathological complete response(p CR)*			
Yes	‐	7(29%)	‐

Abbreviations: 3DCRT, three dimensional conformal radiotherapy; dCRT, definitive chemoradiotherapy; Gy, gray; IMRT, intensity modulated radiotherapy; LFU, lost to followup; N, nodal stage; NACRT, neoadjuvant chemoradiotherapy; NACT, neoadjuvant chemotherapy; RT, radiotherapy; T, tumor stage.

^a^ Chi‐Square Test for categorical variables.

^b^ Mann Whitney Test for continuous variables.

**TABLE 2:** Recurrence, time, and survivals

Site of recurrence	Group 1 dCRT (n = 99)	Group 2 NACRT (n = 24)	*P*‐value
Crude locoregional failure (LRF)	52.5%	25%	.022^a^
	n = 52	n = 6	
Cumulative LRF	64%	35%	.050^b^
Crude local failure (LF)	48.5%	8.3%	.000^a^
	n = 48	n = 2	
Cumulative LF	59%	12%	0.000^b^
Crude regional nodal failure (RLNF)	13%	17%	0.659^a^
	n = 13	n = 4	
Cumulative RLNF	30%	24%	0.592^b^
Crude distant metastasis	18.2%	12.5%	0.763^a^
	n = 18	n = 3	
Cumulative distant metastasis	40%	17%	0.129^b^
5‐Year overall survival median survival (months)	19.6% 6(95% CI:6‐10)	37.5% 24(95% CI:16‐38)	0.014^b^
LRF without concomitant distant metastasis	40.4% n = 40	20.8% n = 5	0.048^a^
Local failure without distant metastasis	39% n = 39	8.3% n = 2	0.001^a^
Regional nodes without distant metastasis	5% n = 5	12.5% n = 3	0.356^a^
Median time to LRF (months)	6.0(1‐81)	20(4‐32)	0.090^c^
Median time to LR (months)	7.65(1–80)	15.3(4‐26)	0.589^c^
Median time to nodal recurrence (months)	12(4.4‐76)	20(7.7‐32.4)	0.785^c^
Median time to distant metastasis (months)	10.8(2.7‐44.5)	13(10‐15.7)	0.703^c^

Abbreviations: CRT, definitive chemoradiotherapy; NACRT, neoadjuvant chemoradiotherapy.

^a^ Chi‐Square Test for categorical variables.

^b^ LogRank Test for cumulative incidence and survivals.

^c^ Mann Whitney Test for continuous variables.

### 3.1 Loco‐regional failure

**FIGURE 1:**
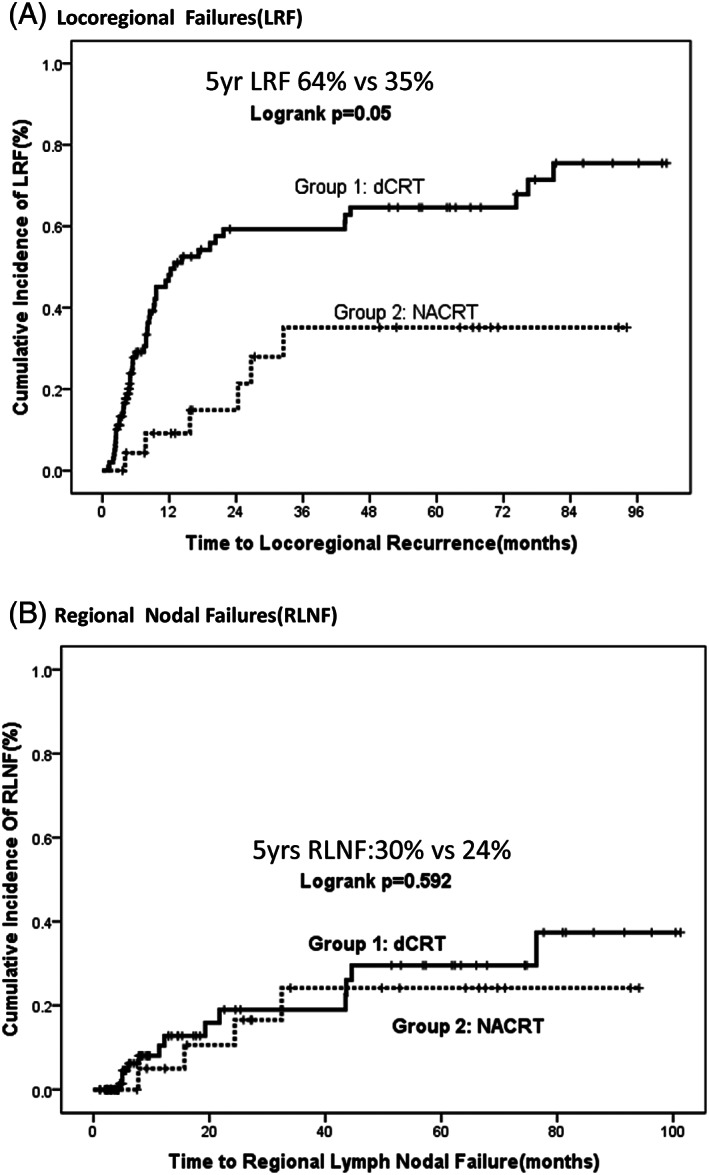
A, Comparison of cumulative incidence of locoregional failures (LRF) between Group 1 (dCRT)and 2 (NACRT). The 5 years cumulative incidence of LRF is 64% vs 35% in Group1 vs 2 (Logrank *P* = .05). B, Comparison of Cumulative incidence of regional nodal failures (RLNF) between Group 1 and 2. The 5 years cumulative incidence of regional lymph nodal failure is 30% vs 24% in Group 1 vs 2 (logrank *P* = .592)

Locoregional failures were documented in 52.5% (n = 52) patients in Group 1 and 25% (n = 6) patients in group 2 (*P* = .022; Figure 1A,B; Tables 2, 3, 4). Cumulative incidence of LRF in Group 1 vs Group 2 at 5 years was 64% vs 35% (*P* = .05) as depicted in Figure 1A. Local failures were documented in 48 patients in Group 1 as opposed to 2 patients in Group 2 (48.5% vs 8.3%, *P* = .000). Local failures were infield in Group 2, and mostly documented as local persistence of disease at primary site in Group 1.

**FIGURE 2:**
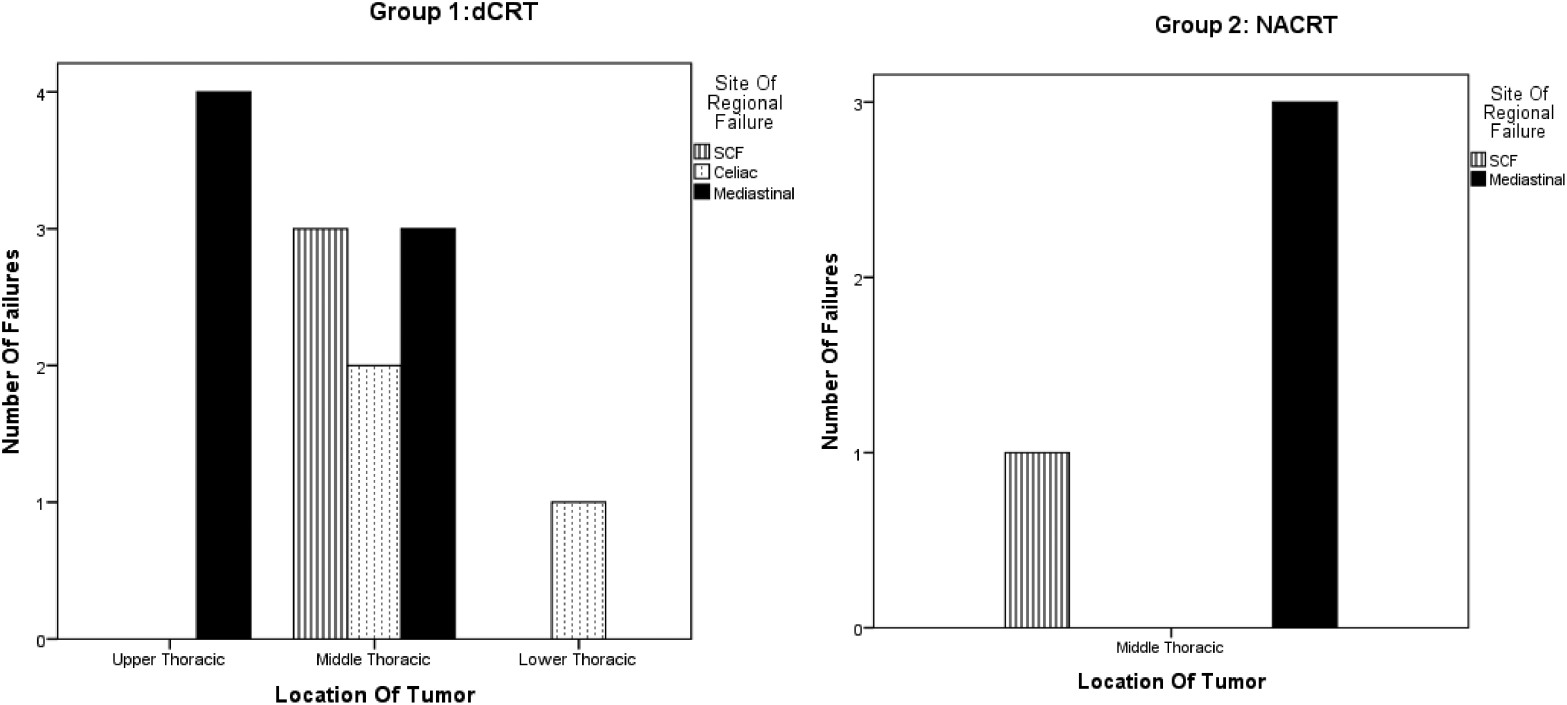
Location of tumor and location of regional nodal failure (in absolute numbers) for Group 1 (dCRT) and Group 2 (NACRT). For both groups mediastinal failures were common

Regional failures were documented in 13 patients in Group 1 as opposed to 4 in Group 2 (13% vs 17%, *P* = .659). Cumulative incidence of RLNF at 5 years was 30% vs 24% in Group 1 vs 2 (*P* = .592) as depicted in Figure 1B. The crude incidence of RLNF without distant metastasis were seen in 5% vs 12.5% (five and three patients in Group 1 and Group 2, respectively), *P* = .356. Most common RLNF in Group 1 was mediastinal (n = 6, 6%) followed by celiac (n = 4, 4%) and SCF (n = 3, 3%). Most common RLNF in Group 2 was mediastinal (n = 3, 12.5%) followed by SCF (n = 1, 4%). Among regional failures, most were out of field in Group 1 (n = 9, 69.2%) located in SCF and celiac regions as compared to Group 2 in which most failures were in field (n = 3, 75%) located in mediastinal regions. Out of field regional failures without distant metastasis were seen in three patients (3/99 = 3%) in Group 1 and one patient (1/24 = 4%) in Group 2. Location of disease had an impact on location of regional failure as depicted in Figure 2.

On univariate analysis, age ≤50 (OR: 2.24, 95% CI: 1.21‐4.19, *P* = .010), incomplete RT (OR: 13.5, 95% CI: 5.77‐31.68, *P* = .00), and absence of concurrent chemotherapy (OR: 2, 95% CI: 1.104‐3.624, *P* = .022) had increasing odds of LRF in Group 1. Dose >50 Gy (OR: 0.148, 95% CI: 0.069‐0.318, *P* = .00) had decreased odds of LRF for Group 1 while absence of comorbidities had decreased odds for LRF in Group 2 (OR: 0.118, 95% CI: 0.019‐0.732, *P* = .023). Age ≤50 (OR: 4.24, 95% CI: 1.23‐14.6, *P* = .022) predicted for RNLR for Group 1, while absence of comorbidities (OR: 0.090, 95% CI: 0.008‐0.993, *P* = .049), were significant for RNLR in Group 2 (Table S1).

On multivariable analysis for LRF, age ≤50 (OR: 3.878, 95% CI: 1.86‐7.84, *P* = .000), incomplete RT (OR: 24, 95% CI: 3.4‐166, *P* = .001), and absence of concurrent chemotherapy (OR: 2.1, 95% CI: 1.019‐4.294, *P* = .0044) retained significance for LRF in Group 1. Absence of comorbidity (OR: 0.013, 95% CI: 0.00‐0.41, *P* = .015) was significant for LRF in Group 2. For RNLR in Group 1, age ≤50 (OR: 4.564, 95% CI: 1.021‐20.48) predicted for increased odds of recurrence. Due to low local, regional failure events in Group 2, multivariable analysis was not done (Table 4).

### 3.2 Distant metastasis

**FIGURE 3:**
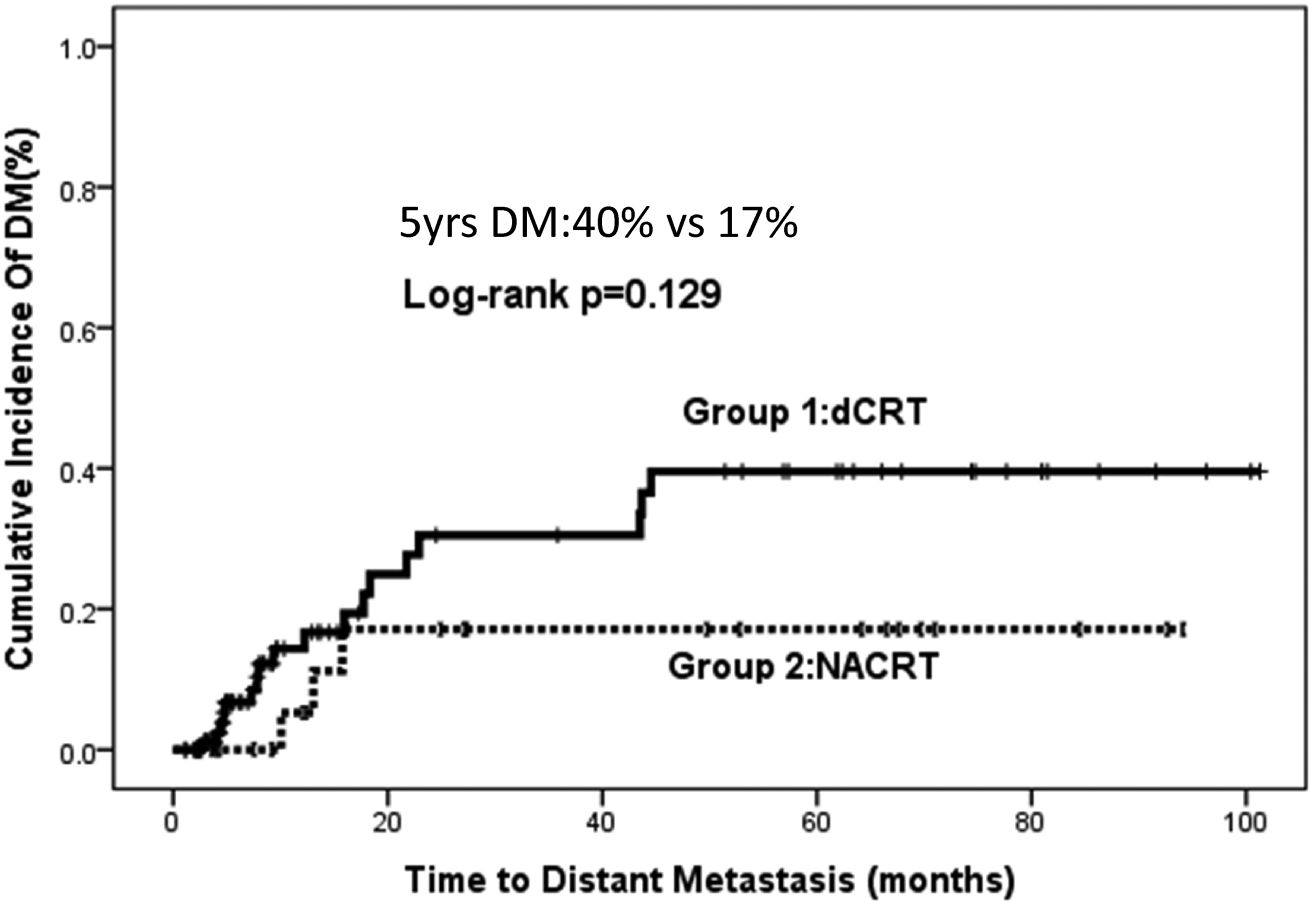
Comparison of cumulative incidence of distant metastasis (DM) between Group 1 (d CRT) and 2 (NACRT). The 5 years cumulative incidence of distant metastasis is 40% vs 17% in Group 1 vs 2 (logrank *P* = .129)

Distant metastasis was documented in 21 and 3 patients in Group 1 and Group 2, respectively (20.6% vs 14.3%, *P* = .763; Figure 3; Tables 3 and 4). The 5 years cumulative incidence of distant failure was 40% vs 17% (*P* = .129) as depicted in Figure 3. The most common site of distant metastasis was lung (5/21 = 28%) in Group 1 as opposed to nonregional lymph nodes in Group 2 (2/3 = 67%). On univariable analysis, age ≤50 (OR: 2.77, 95% CI: 0.99‐7.77, *P* = .0052), incomplete RT (OR:16.5, 95% CI: 2.66‐102.5, *P* = .003), and absence of concurrent chemotherapy (OR: 3.196, 95% CI: 1.2‐8.51, *P* = .020) had increasing odds of DM in Group 1 while none of the factors were significant for Group 2. On multivariable analysis, age ≤50 (OR: 5.15, 95% CI: 1.25‐21.1, *P* = .023), and concurrent chemotherapy (OR: 5.176, 95% CI: 1.542‐17.38, *P* = .008) were significant predictors for Group 1 while for Group 2, due to low events, multivariable analysis was not done (Table 4).

**TABLE 3:** Pattern of regional and distant recurrence

	Group 1 dCRT (n = 99)	Group 2 NACRT (n = 24)
*Regional nodal failure sites*		
Supraclavicular	3(3%)	1(4.1%)
Mediastinal	6(6%)	3(12.5%)
Celiac	4(4%)	0
*Regional nodal failure location*		
Infield regional failures	4, (1 = SCF, 3 = Mediastinal)	3(=Mediastinal)
	4%	12.5%
Out of field regional failures	9, (2 = SCF, 4 = Celiac, 3 = Mediastinal)	1(SCF)
	9%	4%
*Out of field node* × *regional nodal failur*		
Out of field with distant mets	6 (1SCF, 3 = Celiac, 2 = Mediastinal)	0
	(6%)	
Out of field nodal failure without distant metastasis	3 (1 = SCF, 1 = Celiac, 1 = Mediastinal)	1(SCF)
	(3%)	(4%)
*Location of disease* × *Regional Nodal failure*		
Upper thoracic	4 (Mediastinal) (4%)	
Middle thoracic	8 (3 = SCF, 3 = Mediastinal, 2 = Celiac) (8%)	4 (1 = SCF, 3 = Mediastinum) (16.6%)
Lower thoracic	1(=Celiac) (1%)	0
*Location of disease* × *local recurrence*		
Upper thoracic	18(18%)	‐
Middle thoracic	20(20%)	0
Lower thoracic	10(10%)	2(8.3%)
*Distant metastasis sites*		
Lung	5(5%)	0
Liver	3(3%)	0
Peritoneal	3(3%)	0
Non regional nodal	1(1%)	2(8.3%)
Others (multiple sites)	6(6%)	1(4.1%)

*Note*: Infield regional failures: failures located within the radiotherapy portal; Out of Field regional: failures located outside the radiotherapy portal.

Abbreviations: dCRT, definitive chemoradiotherapy;NACRT, neoadjuvant chemoradiotherapy.

**TABLE 4:** Multivariable analysis

Group	Group 1 (dCRT)				Group 2 (NACRT)
Factors	LRF	LR	RLNR	DM	LRF
*Age*					
≤50	**0.000**	**0.001**	**0.047**	**0.0023**	0.417
>50 (Ref)	HR:3.878	HR:3.493	HR:4.564	HR:5.15	HR:0.215
	(1.86–7.84)	(1.628‐7.498)	(1.021‐20.48)	(1.25‐21.1)	(0.005‐8.71)
*Gender*					
Male	0.796	0.832	0.138	0.161	0.176
Female (Ref)	HR:1.094 (0.556‐2.152)	HR:0.926 (0.457‐1.877)	HR:3.751 (0.053‐21.55)	HR:2.585 (0.68‐9.73)	HR:11.25 (0.337‐376.12)
*Comorbidities*					
Present (Ref)	0.362	0.120	0.473	0.332	**0.015**
Absent	HR:0.750 (0.404‐1.392)	HR:0.596 (0.311‐1.143)	HR:1.665 (0.4149‐6.699)	HR:1.86 (0.53‐6.55)	HR:0.013 (0.00‐0.42)
*Length*					
>5 cm (Ref)	0.285	0.258	0.303	0.46	
≤5 cm	HR:1.490 (0.718‐3.092)	HR:1.562 (0.721‐3.380)	HR:2.201 (0.491‐9.872)	HR:0.617 (0.168‐2.259)	HR:1.259 (0.073‐21.66)
*Location*	0.935				
Upper	HR:0.966 (0.424‐2.201)	0.666 HR:1.209 (0.511‐2.862)	0.924 HR:1.118 (0.112‐11.11)	0.073 HR:0.293 (0.07‐1.12)	‐
Middle	0.282	0.433	0.667	0.111	0.915
Lower (Ref)	HR:0.653 (0.301‐1.419)	HR:0.720 (0.316‐1.630)	HR:1.649 (0.169‐16.08)	HR:0.342 (0.091‐1.282)	HR:0.859 (0.005‐14.03)
*NACT*	0.568	0.505	0.750	0.462	0.478
Yes (Ref)	HR:1.308 (0.520‐3.291)	HR:1.404 (0.518‐3.801)	HR:0.748 (0.125‐4.454)	HR:1.845 (0.361‐9.426)	0.454 (0.051‐4.036)
No					
*Concurrent CT*	**0.044**	**0.004**	0.378	**0.008**	0.993
Yes (Ref)	HR:2.092 (1.019‐4.294)	HR:2.126 (1.004‐4.49)	HR:1.897 (0.456‐7.803)	HR:5.176 (1.542‐17.38)	HR:0.04 (0.0‐50 195)
No					
*Dose*	0.597	0.278	0.991	0.206	‐
≤50 (Ref)	HR:0.609 (0.097‐3.828)	HR:0.418 (0.086‐2)	HR:0.00 (0.00‐100)	HR:4.79 (0.423‐54.34)	
>50					
*RT Complete*	**0.001**	**0.000**	1	0.301	‐
Yes (Ref)	HR:24 (3.4‐166)	HR:40.06 (6.86‐234.02)	HR:3.386 (0.00‐ > 100)	HR:4.389 (0.266‐72.49)	
No					

*Note*: The bold values indicate statistically significant values.

Abbreviations: CT, chemotherapy; dCRT, definitive chemoradiotherapy; DM, distant metastasis; LF, local failure; LRF, locoregional failure; NACRT, neoadjuvant chemoradiotherapy; NACT, neoadjuvant chemotherapy; RLNF, regional lymph nodal failure; RT, radiotherapy.

### 3.3 Overall survival

The 5‐year OS in Group 1 was 19.6% vs 37.5% in Group 2 with median survival of 6 months for Group 1 (95% CI 6‐10) vs 27 months for Group 2 (95% CI 16‐38), *P* = .014 (Table 2).

## 4 DISCUSSION

The management of localized or locally advanced esophageal carcinoma is difficult because of the high likelihood of loco‐regional recurrences. In this study, we found that locoregional disease was the predominant reason for treatment failure in both groups(Figure 1A), and the crude incidence of LRF was significantly high in Group 1 vs Group 2 (52.5% vs 25%, *P* = .022). These results were no different from other studies suggesting that the LRF is a common mode of relapse after NACRT or dCRT.^13^, ^14^, ^15^ Locoregional failure without distant metastasis was seen in nearly half the patients in Group 1 (49.4%) as opposed to one‐fourth in Group 2 (25.4%). As in our study, LRR rates of 13% to 25%^16^, ^17^ are reported after NACRT as compared to 43% to 55%^18^ in patients receiving dCRT. Isolated local failure without concomitant distant metastasis was seen in 39 and 2 patients (39% vs 8.3%, *P* = .001). Local failures were common in Group 1 as opposed to regional nodal failures in Group 2. The crude and cumulative incidence of failures is depicted in Table 2.

The evidence underlying RT planning volumes, in particular CTV, is poorly defined, and very little published data are available regarding the margins required to allow for microscopic disease extension from the defined gross target volume (GTV).^7^, ^19^ In published series for dCRT, the margins have varied from elective treatment of the whole esophagus with the SCF to a defined 3 to 5 cm SI margin above and below the defined GTV.^20^ For preoperative CRT, a fixed margin above and below the GTV has been used in trials,^20^ but controversy exists regarding the use of prophylactic nodal radiation.^19^ The esophageal submucosa has an extensive lymphatic vertical distribution, and numerous studies have suggested that multiple levels and skipped node metastases are commonly observed in esophageal SCC.^21^, ^22^ As per our analysis, RLNF rates without distant metastasis were seen in 5% vs 12.3% in Group 1 vs Group 2, *P* = .356 (five and three patients, respectively, Table 3). These nodal failures in both groups were most commonly located in the upper mediastinal nodal regions, similar to the reported site of failures in surgical series of thoracic ESCC.^23^, ^24^ Nakagawa et al^25^ reported that for thoracic ESCC, the lymph node metastasis of cervical and upper mediastinal lymph nodes is higher. In addition, there was an impact of tumor location on the regional nodal failure (Figure 3). In Group 1 patients, for upper esophagus, mediastinal failures were common, and SCF failures were uncommon probably due to prophylactic irradiation of SCF using a T‐shaped field in these patients. However, for middle esophagus, failures were seen in both SCF, celiac as well as mediastinal nodal regions. Lower esophagus location in Group 1 had regional failures in the celiac region. The bidirectional transfer probability of middle thoracic esophageal carcinoma is higher as reported in surgical studies as well.^21^, ^26^ .The regional failures in Group 2 patients for disease in middle esophagus were seen in the high mediastinal region while for lower esophagus no regional failures were documented. Studies have pointed out that invasion depth, differentiation, and lesion length are correlates with lymph node metastasis in esophageal cancer.^27^, ^28^, ^29^ Length of disease was not a significant predictor of RNLR for both groups on uni/multivariable analysis. There are limited data in the literature regarding the impact of the extent of lymphadenectomy on outcomes in patients receiving multimodality therapy in thoracic esophageal SCC esophagus. For patients in Group 2, whether a correlation exists between RLNF and field of lymph node dissection is outside the scope of this analysis, and it requires further evaluation as nodal failures were commonly seen in mediastinum, and most of these mediastinal failures were in the high mediastinum outside the regular two field dissection. Cervical lymphadenectomy was not routinely performed in our cancer center as studies have clearly shown an increased risk of mortality with extensive lymph node dissection.^30^

Regional nodal failures were mostly out of field in the dCRT (n = 9/13, 69%) group as compared to being infield in the NACRT (n = 3/4, 75%) group. Eloubeidi et al^29^ reported that longer lesions led to more lymph node metastasis outside the target volume, and as patients with dCRT had a greater length, most failures were out of field. Overall isolated out‐of‐field regional failure without distant metastasis in the nontreated elective regions was only 3% to 4%, in both the groups, similar to the rates of 3% reported by Button et al,^7^ and it is unlikely that treating additional lymph node regions would have a meaningful clinical impact as the micrometastases at distant sites would nullify the benefits of clearance of locoregional disease. Nevertheless, local failures in both the dCRT and NACRT were infield. These results further support focusing the attention of local therapies on improving in‐field control than extending fields, to include prophylactic volumes.^31^

Despite the negative findings of INT01232, a number of studies have suggested a potential benefit for dose escalation.^31^, ^32^, ^33^ Local failure (without distant metastasis) rates of 39% in Group 1 patients (majority T3/4 and node positive) are promising and support a rationale to revisit dose escalation. A recent study in appropriately staged patients with PET/CT reported similar failure rates of 39% in patients treated with high dose dCRT.^34^ In the current study, dose of RT ≤50 Gy was received by 17% of patients in dCRT, and it was a significant predictor on univariable analysis for LRF and LF but failed to retain significance on multivariable analysis. Most patients receiving ≤50 Gy (n = 17) in the dCRT group did not complete RT (13/17 = 76%) due to reasons of noncompliance (nine patients) and death (four patients), with these observations considered as events for LRF/LF and thereby incomplete RT rather than dose, retained significance on multivariable analysis. Ongoing trials are testing dose escalation in esophageal SCC using modern radiation techniques.^34^, ^35^Despite modest dose escalation, the local failure rates were high. Although it is important that the RT technique is optimized through ongoing research, given the high cumulative rates of distant metastasis in Group 1, it is very likely that improvements in outcome will result from the use of effective systemic therapy, producing better radiosensitization for local control and systemic antitumor response. Neoadjuvant chemotherapy has produced modest survival gains before surgical therapy in esophagogastric cancer,^36^ suggesting that early systemic therapy might be capable of sterilizing occult disease and/or making local therapy more effective.^37^ The use of NACT before RT debulks tumor, provides dysphagia relief, and it is a common practice in the United Kingdom with neoadjuvant regimens being tested in trials.^38^ In our study, 10% to 15% of patients received NACT in both groups, and it did not impact on locoregional or distant metastasis. On the other hand, use of concurrent chemotherapy improved locoregional control and distant metastasis both in univariate and multivariable analysis for Group 1. We used single agent Cisplatin chemotherapy @ 35 mg/m^2^, given as a weekly regimen, for reasons of feasibility and better tolerance in view of the extensive length of disease at presentation. Platinum‐containing drugs, fluoropyrimidines, and taxanes are all established as concurrent systemic agents, and evidence from trials of dCRT or NACRT have used a dual agent with platinum backbone in combination with taxane or 5 Fluorouracil.^39^ The 5‐year crude incidence of distant recurrence was, in fact, similar between groups (20.6% for dCRT vs 14.3% for NACRT). The 5‐year cumulative incidence of distant metastasis, although numerically higher, was statistically not significant between groups (40% for dCRT vs 17% for NACRT,), potentially owing to follow‐up time and/or sample size issues. The distant failure rate of 40%, as first site of relapse, in Group 1 was due to higher stage disease in our patient population, and, although similar to 39% as reported by Effeny et al,^34^ it highlights the need for improved (dual agent) systemic therapies.

Young age (≤50 years) predicted for higher odds of LRF, RNLF, and DM for dCRT, and these patients require aggressive treatment approaches. For Group 1, median time to any failure was within a year (6‐12 months), whereas it was within 2 years in Group 2 (25‐29 months) suggesting surveillance strategies should be intensive for the first 2 to 3 years. This finding validates our follow‐up policy. The 99 patients undergoing dCRT were more likely to have SCC and locally advanced tumors with two thirds of patients having length of disease >5 cm than patients undergoing surgery. As expected, OS was significantly better in Group 2 (Table 2) with median survival in Group 1 of 6 months vs 27 months in Group 2 (*P* = .014). There has been a debatable role of surgery in SCC esophagus, but it is also well known that surgery decreases the rate of malignant dysphagia. This effective local treatment by reducing the LRF impacts on OS, and despite imbalances between groups, is suggested from our analysis as well.

This study has several limitations. First, it is a retrospective study wherein an observational comparison of two treatment modalities was done with possibility of inherent selection biases. Consequently, the groups were unbalanced in terms of length and location of disease. Second, PET staging was not done at baseline, and some patients could be metastatic at presentation as suggested by studies that PET/CT can exclude an additional 10% to 15% of patients with distant metastasis.^40^ All recurrences, especially after definitive CRT, were not confirmed on pathology, which may influence the accuracy of recurrence information. Additionally, all patients at the time of developing local recurrence in the dCRT did not undergo a CECT scan, which could have underestimated the nodal failures, especially in the mediastinum. Further, there were 20 patients lost to follow‐up in dCRT, and 1 patient in NACRT immediately after completion of treatment. However, the strength of this study lies in the fact that it represents a consecutive patient population treated by a multidisciplinary team. Also, the follow‐up investigation practices based on symptomatic progressions are pragmatic and representative of routine clinical practice.

In conclusion, LRF are a frequent occurrence both in dCRT and NACRT with RLNF being common in NACRT as opposed to LF in dCRT. Among regional nodes, mediastinal nodal failure was the most common in both the groups. With respect to field design, regional failures in dCRT were out of field, but most of them had concurrent distant metastasis. Conversely, regional failures in the NACRT group were infield (high mediastinum), and without concurrent distant metastasis. Concurrent chemotherapy enhances locoregional control and decreases distant metastasis in patients undergoing dCRT while young age predicts for increased LRF and DM. Modification of RT fields would unlikely be helpful. Optimization of systemic therapy for dCRT and field of dissection for NACRT might be warranted.

## ACKNOWLEDEMENTS

None.

## CONFLICT OF INTEREST

The authors have stated explicitly that there are no conflicts of interest in connection with this article.

## AUTHOR CONTRIBUTIONS

All authors had full access to the data in the study and take responsibility for the integrity of the data and the accuracy of the data analysis. *Guarantor of integrity of the entire study*, R.K.S., S.K., S.M.; *Study concepts and design*, S.M., F.A., S.S., R.K.S., K.J.M.D.; *Literature research*, S.M., F.A., S.K.; *Clinical studies*, F.A., S.K., S.M., S.S., K.J.M.D.; *Data analysis*, S.M., R.K.S., S.K.; *Statistical analysis*, S.K., S.S., S.M., R.K.S.; *Manuscript preparation*: S.M., F.A., S.S., K.J.M.D.; *Manuscript editing*, R.K.S., F.A., S.K., S.M., S.S., K.J.M.D.

## ETHICAL STATEMENT

The institutional review board approved this study (approval number 2019‐119‐IP‐EXP‐9 at SGPGIMS, Lucknow, India).

## Supporting information


**Supplementary Figure S1** Schematic of patient inclusion
**Supplementary Figure S2**: Radiotherapy planning in Phase 1 and 2 with DRR, 95% Isodose colorwash and description of target volumes as depicted in table.

**Supplementary Table S1** Univariable Analysis

## Data Availability

The data have not been submitted but will be available on request to the corresponding author.
