# Supplementary material for: Assessing failure patterns of radical intent radiation strategies in patients with locally advanced carcinoma of the esophagus
Source: Cancer Rep (Hoboken). 2020 Dec 28;4(3):e1332. doi: 10.1002/cnr2.1332 (PMC8222558; doi:10.1002/cnr2.1332)
Supplement: Supplementary file 1 — Supplementary Figure S1 Schematic of patient inclusion Supplementary Figure S2: Radiotherapy planning in Phase 1 and 2 with DRR, 95% Isodose colorwash and description of target volumes as depicted in table. [file CNR2-4-e1332-s001.pptx]

## Slide 1
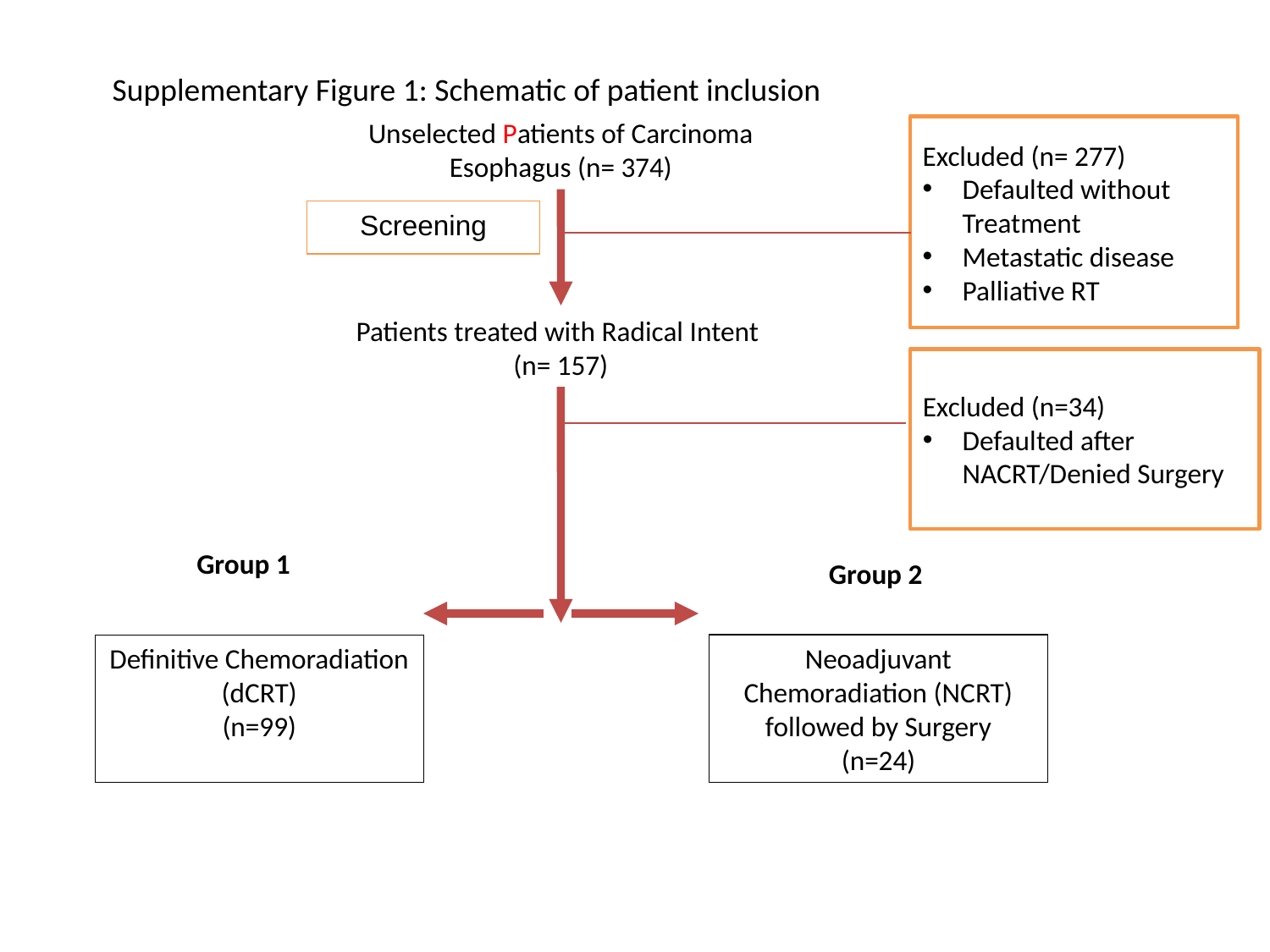

Supplementary Figure 1: Schematic of patient inclusion
Unselected Patients of Carcinoma Esophagus (n= 374)
Excluded (n= 277)
Defaulted without Treatment
Metastatic disease
Palliative RT
Screening
Patients treated with Radical Intent
(n= 157)
Excluded (n=34)
Defaulted after NACRT/Denied Surgery
Group 1
Group 2
Definitive Chemoradiation (dCRT)
(n=99)
Neoadjuvant Chemoradiation (NCRT) followed by Surgery
(n=24)

## Slide 2
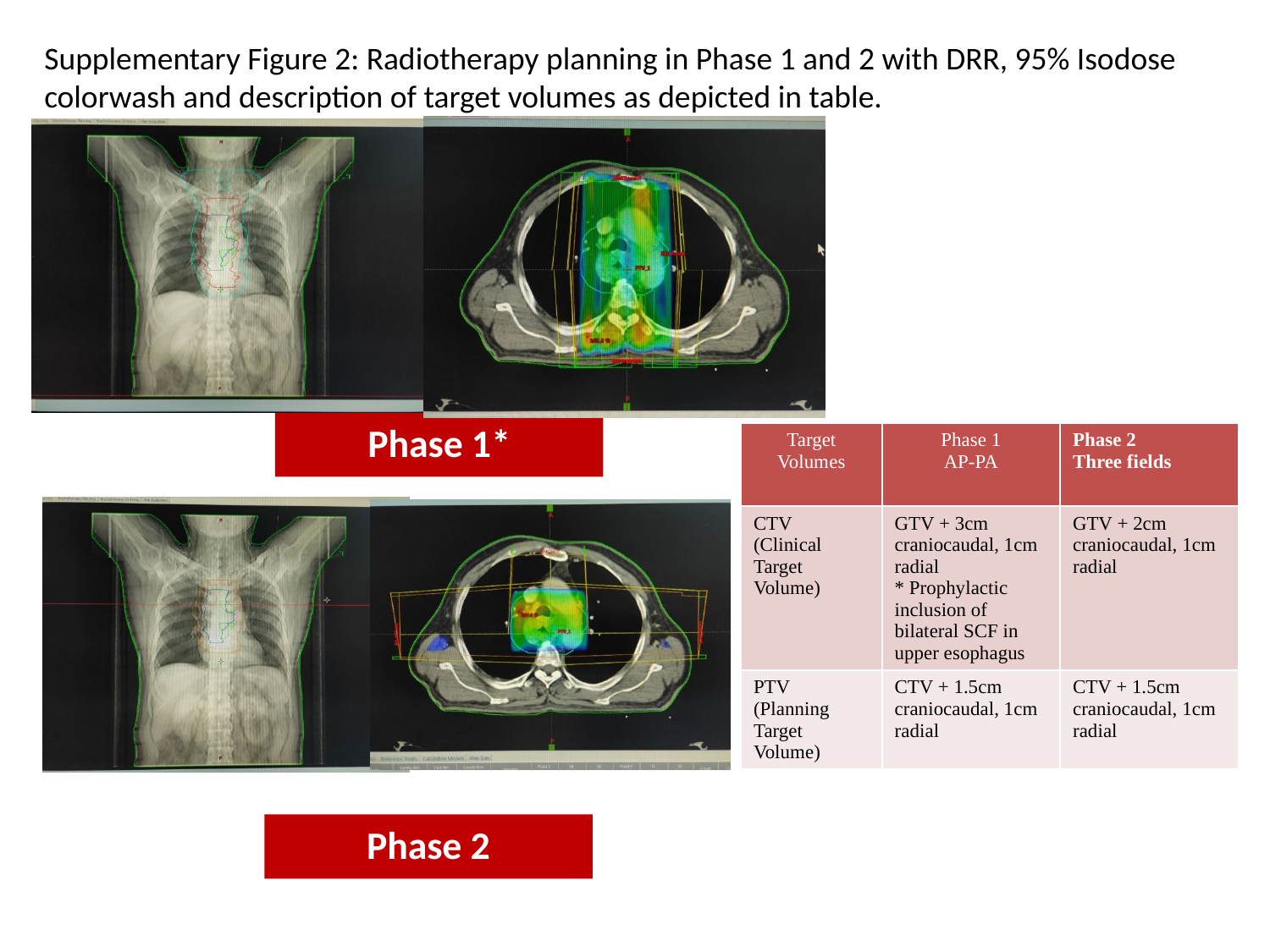

Supplementary Figure 2: Radiotherapy planning in Phase 1 and 2 with DRR, 95% Isodose colorwash and description of target volumes as depicted in table.
Phase 1*
| Target Volumes | Phase 1 AP-PA | Phase 2 Three fields |
| --- | --- | --- |
| CTV (Clinical Target Volume) | GTV + 3cm craniocaudal, 1cm radial \* Prophylactic inclusion of bilateral SCF in upper esophagus | GTV + 2cm craniocaudal, 1cm radial |
| PTV (Planning Target Volume) | CTV + 1.5cm craniocaudal, 1cm radial | CTV + 1.5cm craniocaudal, 1cm radial |
Phase 2
