# Supplementary material for: Assessing failure patterns of radical intent radiation strategies in patients with locally advanced carcinoma of the esophagus
Source: Cancer Rep (Hoboken). 2020 Dec 28;4(3):e1332. doi: 10.1002/cnr2.1332 (PMC8222558; doi:10.1002/cnr2.1332)
Supplement: Supplementary file 2 — Supplementary Table S1 Univariable Analysis [file CNR2-4-e1332-s002.docx]

**Supplementary Table 1. Univariable Analysis**

|  | **Group 1(dCRT)** | | | | **Group 2(NACRT)** | | | |
| --- | --- | --- | --- | --- | --- | --- | --- | --- |
| **Factors** | **LRF** | **LR** | **RLNR** | **DM** | **LRF** | **LR** | **RLNR** | **DM** |
| Age  ≤50  >50 ( Ref) | **0.010**  HR:2.24  (1.21-4.19) | 0.07  HR:1.79 (0.95-3.39) | **0.022**  HR:4.24 (1.23-14.6) | **0.052**  HR:2.775 (0.991-7.772) | 0.778  HR:0.78  (0.14-4.29) | 0.698  HR:1.732 (0.10-27.7 | 0.637  HR:0.58 (0.60-5.5) | 0.865  HR:0.812  (0.074-8.96) |
| Gender  Male  Female (Ref) | 0.494  HR:1.224 (0.685-2.187) | 0.695  HR:1.128 (0.618-2.05) | 0.121  HR:3.299 (0.731-14.891) | 0.196  HR:2.082 (0.685-6.329) | 0.567  HR:1.875 (0.218-16.139) | 0.623  HR:31.3 (0-2831) | 0.961  HR:1.058 (0.109-10.305) | 0.486  HR37.20 (0.001-9758) |
| Comorbidities  Present(Ref)  Absent | 0.794  HR:0.930 (0.538-1.606) | 0.365  HR:0.76 (0.43-1.35) | 0.330  HR:1.799 (0.552-5.869) | 0.200  HR:1.965 (0.699-5.522) | **0.023**  HR:0.118 (0.019-0.732) | 0.203  HR:0.15 (0.008-2.76) | **0.049**  HR:0.090 (0.008-0.993) | 0.355  HR:0.321 (0.029-3.556) |
| Habits  Present(Ref)  Absent | 0.178  HR:0.661 (0.362-1.207) | 0.136  HR:0.615 (0.324-1.166) | 0.225  HR:0.448 (0.123-1.639) | 0.160  HR:0.45 (0.148-1.372) | 0.814  HR:0.825 (0.165-4.119) | 0.474 HR:0.016 (0.00-1381) | 0.421  HR:2.544 (0.261-24.751) | 0.809 HR:1.346 (0.122-14.89) |
| Length  >5cm(Ref)  ≤5cm | 0.898  HR:1.042(0.556-1.954) | 0.955  HR:1.019 (0.529-1.961) | 0.626  HR:1.341 (0.412-4.367) | 0.797  HR:0.864 (0.284-2.629) | 0.435  1.894 (0.380-9.433) | 0.611  HR:2.054 (0.128-32.9) | 0.458  HR:2.104 (0.295-14.997) | 0.499  HR:0.028 (0.00-897.5) |
| Location  Upper  Middle  Lower (Ref) | 0.448  HR:0.749 (0.354-1.581)  0.717  HR:0.874(0.423-1.808) | 0.648  HR:0.83 (0.38-1.8)  0.807  HR:0.90(0.42-1.948) | 0.774  HR:1.38 (0.153-12.46)  0.30  HR:3.012(0.375-24.19) | 0.097  HR:0.347 (0.099-1.213)  0.438 HR:0.642(0.209-1.970) | -  2.041(0.373-11.1) | 0.495  HR:0.019(0.00-1765.4) | 0.296  HR:73.3 (0.02-232690) | 0.547  HR:0.547 (0.049-6.067) |
| Wt loss  ≤10%(Ref)  >10% | 0.807  HR:1.071 (0.617-1.861) | 0.825  0.937 (0.527-1.667) | 0.575  HR:0.731 (0.245-2.185) | 0.957  HR:1.027 (0.397-2.655) | 0.551  0.614 (0.124-3.048) | 0.659  1.871 (0.116-30.26) | 0.572  HR:1.76 (0.267-12.538) | 0.444  HR:0.023 (0.00-362.28) |
| NACT  Yes (Ref)  No | 0.444  HR:1.395 (0.595-3.272) | 0.361  HR:1.54 (0.61-3.89) | 0.864  HR:1.141 (0.252-5.176) | 0.553  HR:1.563 (0.358-6.820) | 0.178  0.332 (0.067-1.655) | 0.483  HR:0.002 (0.00-60332) | 0.993  HR:0.99 (0.102-9.619) | 0.155  HR:0.176 (0.016-1.936) |
| Concurrent  Yes (Ref)  No | **0.022**  HR:2(1.104-3.624) | **0.022**  HR:2.04(1.1-3.77) | 0.507  HR:1.562 (0.418-5.8) | **0.020**  HR:3.196 (1.2-8.51) | 0.661  HR:0.04 (0.0-50195) | 0.814  HR:0.045 (0.00-7477) | 0.726  HR:0.045 (0.00-160680) | 0.773  HR:0.045 (0.00-597) |
| Dose  ≤50(Ref)  >50 | **0.00**  HR:0.148 (0.069--0.318) | **0.00**  HR:0.189 (0.091--0.390) | 0.707  HR:21.68 (0.00->100) | **0.001**  HR:0.083 (0.018-0.374) | - |  | - |  |
| RT Complete  Yes ref  No | **0.00**  HR:13.5 (5.77-31.68) | **0.00**  HR:15.39 (6.33-37.39) | 0.839  HR:0.048 (0.00->100 | **0.003**  HR:16.51 (2.66-102.524) | - | - | - |  |
| pCR  Yes Ref  No |  |  |  |  | 0.186  HR:4.324 (0.494-37.86) | 0.817  HR:0.714 (0.041-12.42) | 0.350  HR:50 (0.014->100) | 0.416  HR:50 (0.004->100) |

Wt:Weight;NACT:Neoadjuvant Chemotherapy;RT:Radiotherapy; p CR: pathological complete response;LRF:Locoregional Failure;LF: Local Failure;RLNF:Regional lymph nodal failure;DM: Distant Metastasis;dCRT:Definitive Chemoradiotherapy;NACRT:Neoadjuvant Chemoradiotherapy
